# Supplementary material for: Function of histone H2B monoubiquitination in transcriptional regulation of auxin biosynthesis in Arabidopsis
Source: Commun Biol. 2021 Feb 15;4:206. doi: 10.1038/s42003-021-01733-x (PMC7884795; doi:10.1038/s42003-021-01733-x)
Supplement: Supplementary file 2 — Supplementary Information [file 42003_2021_1733_MOESM2_ESM.pdf]

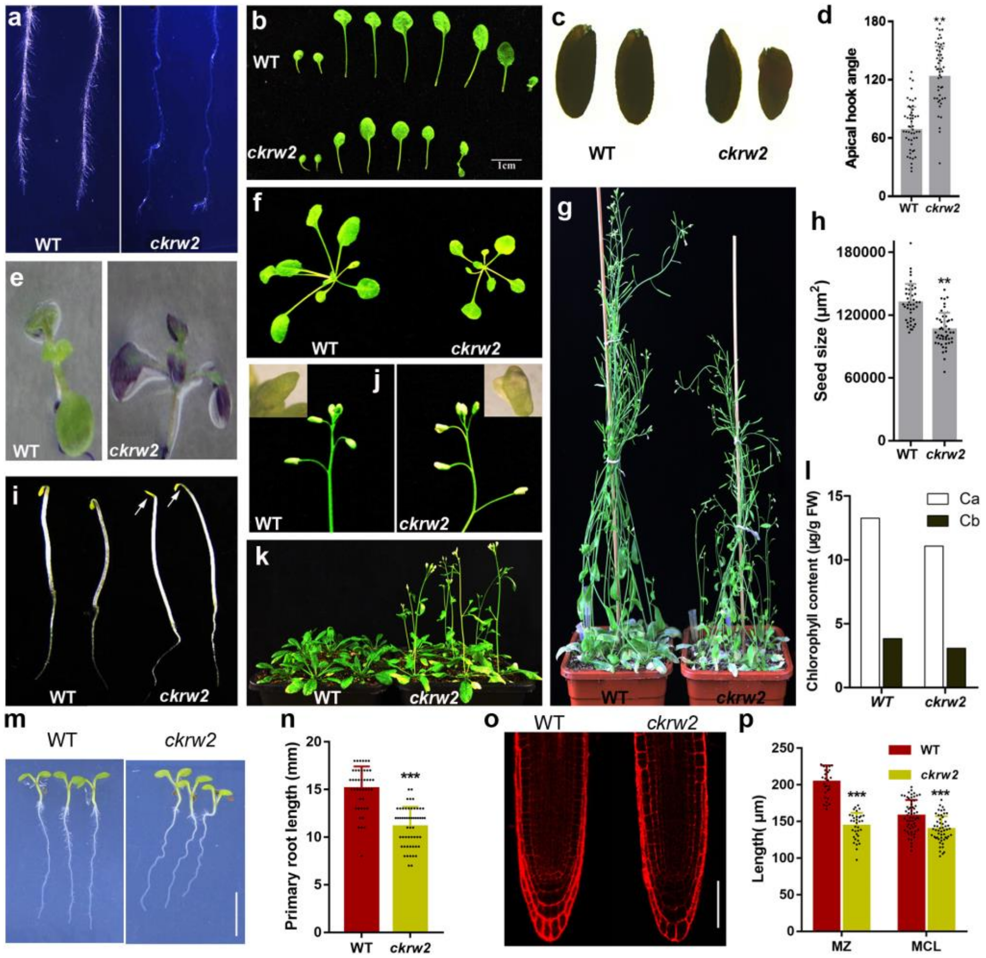

Supplementary Figure 1. Pleiotropic phenotypes of *ckrw2* mutant, showing significant differences between WT and *ckrw2* in root hairs (**a**), leaves (**b**), seed size (**c**), cutin (**e**), seedling size (**f**), plant height (**g**), apical hook (**i**), petals (**j**) and flowering time (**k**). **m**, The phenotype of *ckrw2* on MS medium. **o**, The root cell structure revealed by PI staining. Data in statistical analyses shown in **d**, **h**, **l**, **n** and **p** are presented as mean ± SD (n=35-50) from three independent biological experiments, the asterisk indicates a significant difference based on Student's t test with $^{**}P<0.01$, $^{***}P<0.001$.

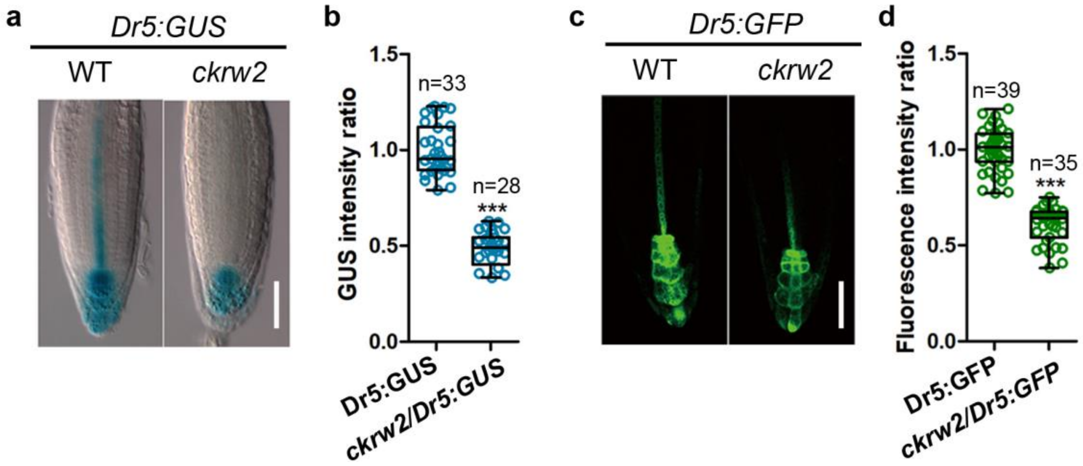

Supplementary Figure 2. Auxin activity in root tips revealed by the expressions of Dr5: GUS/GFP, respectively. The seedlings grown on MS medium for 7 days were used for GUS staining and fluorescence intensity observation (approximately to the first 200 µm from the root tip). Each circle represents the measurement from an individual root. Boxplots span the first to the third quartiles of the data. Whiskers represent minimum and maximum values. A line in the box represents the mean. "n" represents the number of roots used in this experiment. Student's t-test, was used for statistical analyses. $^{***}P < 0.001$. Bars=50 µm in (a, c).

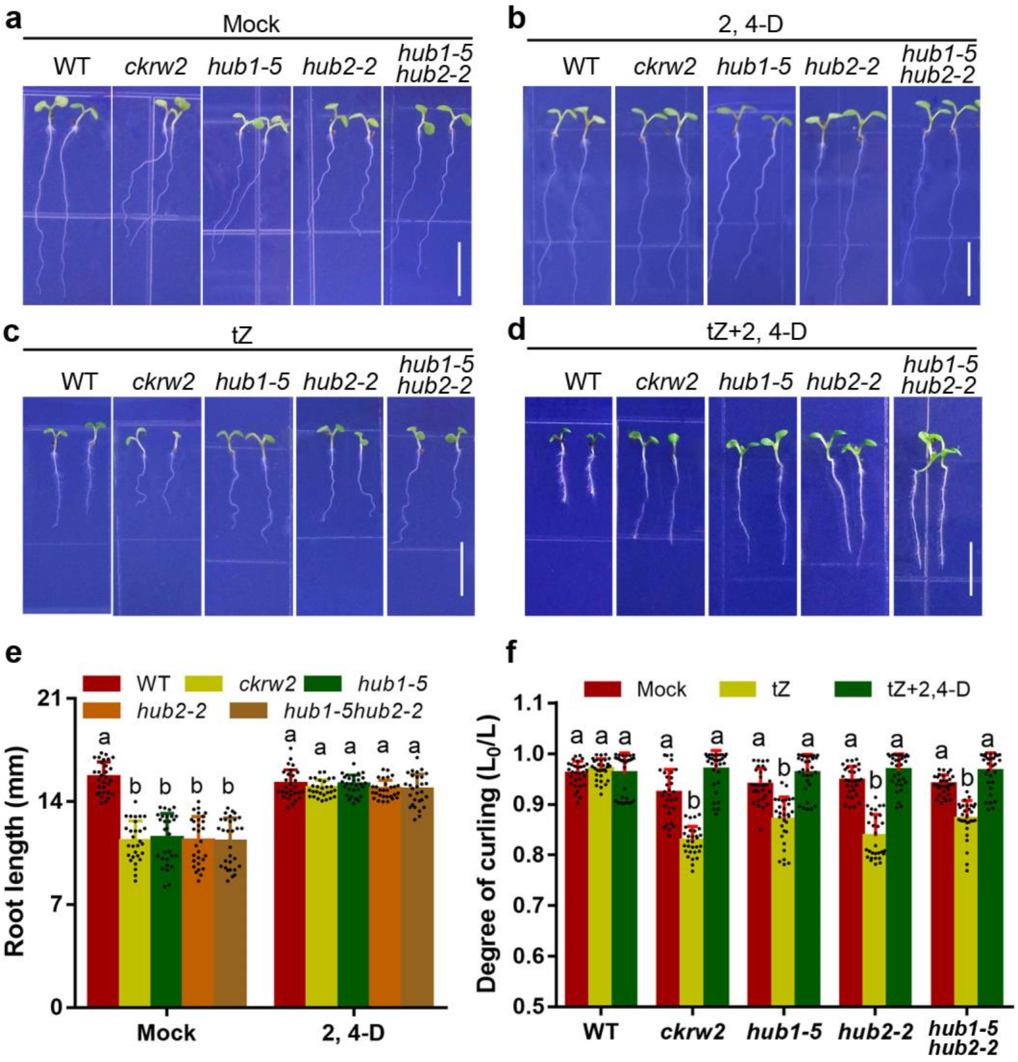

Supplementary Figure 3. Phenotypic characterization of *ckrw2* and other *hub1* allelic mutants. Plantlets were grown on the MS media without hormone (**a**), or with 0.01 μM 2, 4-D (**b**) or 0.01 μM tZ (**c**) or both (**d**) for 7 days. Bars=5 mm in (a-d). Data shown in **e** and **f** are presented as mean ± SD (n=30-35), three independent experiments, the letters indicate a significant difference at P<0.05, according to ANOVA followed by Tukey's multiple comparison tests.

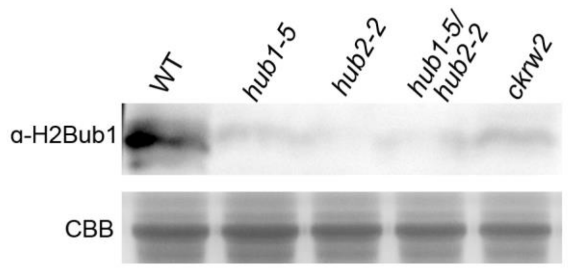

Supplementary Figure 4. H2Bub1 levels in WT and *hubs*. CBB, Coomassie brilliant blue-stained RbcL protein, loading control. α-H2Bub1, anti-Histone H2B monoubiquitinated antibody.

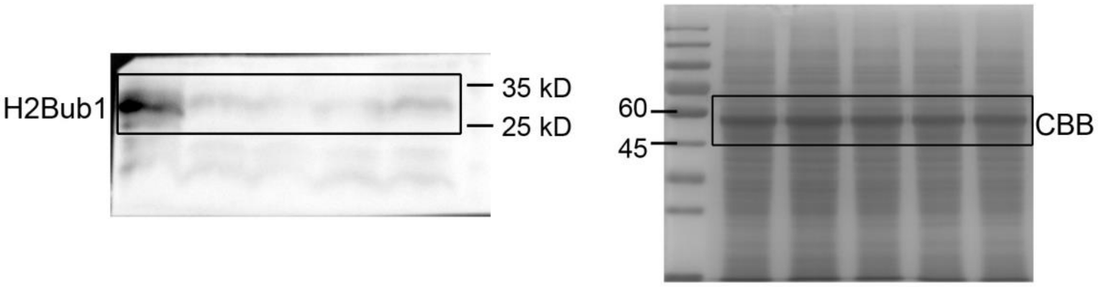

Supplementary Figure 5. Uncropped Western-blots images corresponding to Supplementary Figure 4.

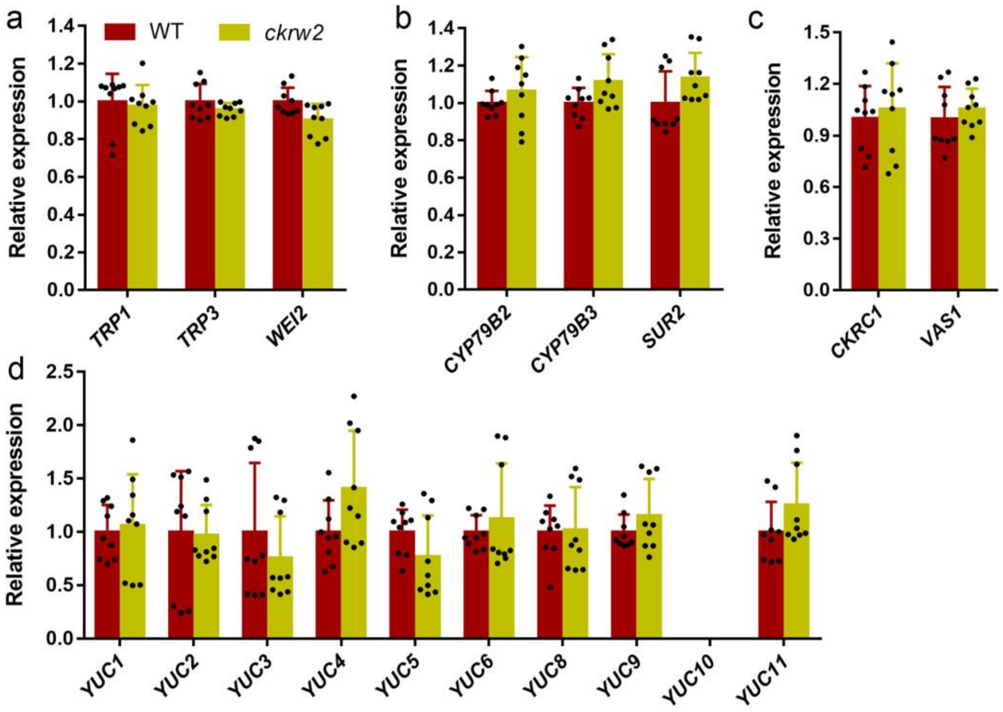

Supplementary Figure 6. Expression of related genes in the auxin synthesis pathway. Data are presented as mean ± SD, three independent experiments, the asterisk indicates a significant difference between the *ckrw2* mutant and WT based on Student's t test ($^{**}P<0.01$). ACTIN8 was used as an internal control.

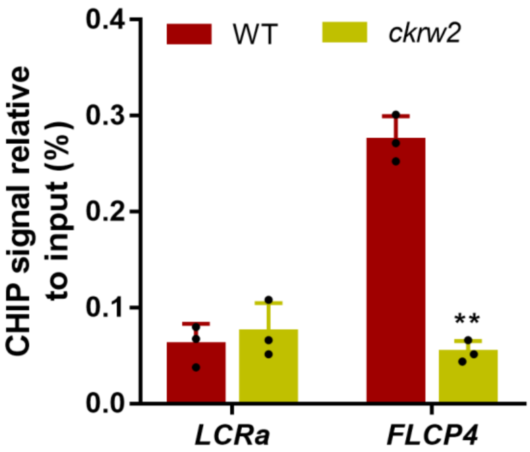

Supplementary Figure 7. ChIP analysis of H2B monoubiquitin deposition at specific loci. *LCRa* and *FLCP4* were used as negative and positive controls, respectively. Data are presented as mean ± SD, three independent biological experiments, the asterisk indicates a significant difference based on Student's t test with $^{**}P<0.01$.

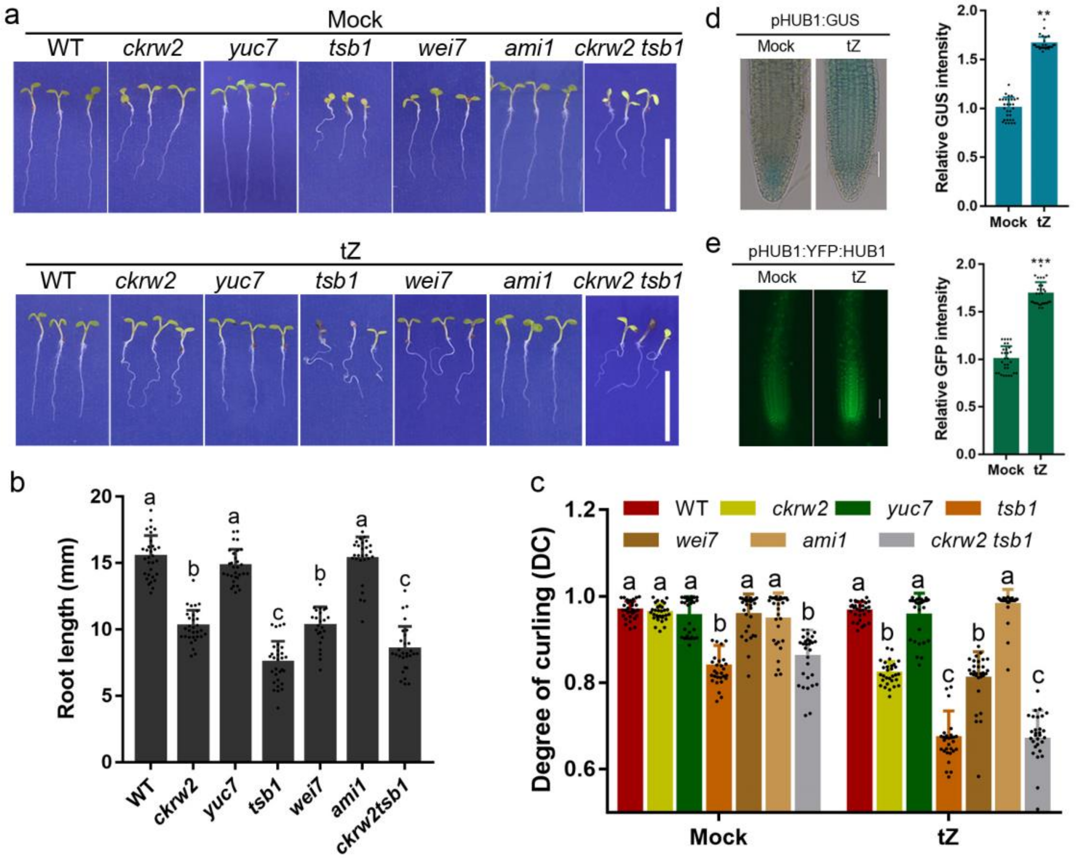

Supplementary Figure 8. Phenotypic observation and genetic analysis of *ckrw2* and the four of its related mutants (a-c), and the CK-induced *HUB1* gene expression (d,e). The tZ concentration used was 0.01 μM. Data are presented as mean ± SD, three independent experiments, the letters (b, c) indicate a significant difference at P<0.05, according to ANOVA followed by Tukey's multiple comparison tests, and the asterisk (d, e) indicates a significant difference based on Student's t test (**P<0.01, ***P<0.001).

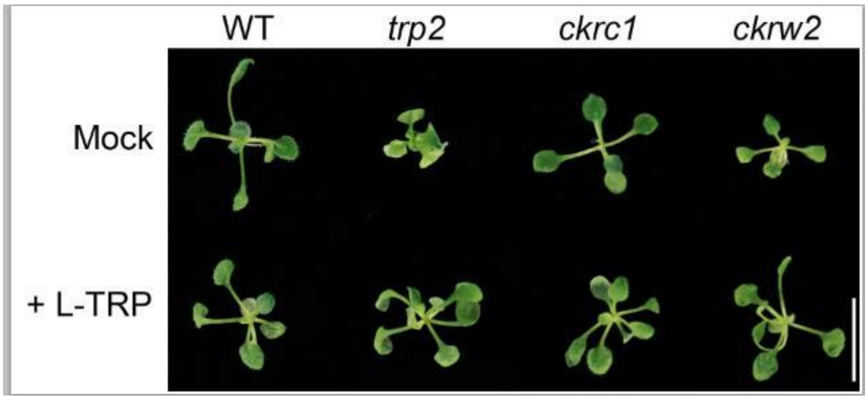

Supplementary Figure 9. Comparison of the rescuing effects of L-Trp on 3-week-old plants between WT, *ckrw2, trp2* and *ckrc1* mutants. The 1-week-old seedlings germinated on MS plates were transferred to fresh MS plates with or without 0.25 mM L-Trp, and were further grown for 2 weeks.

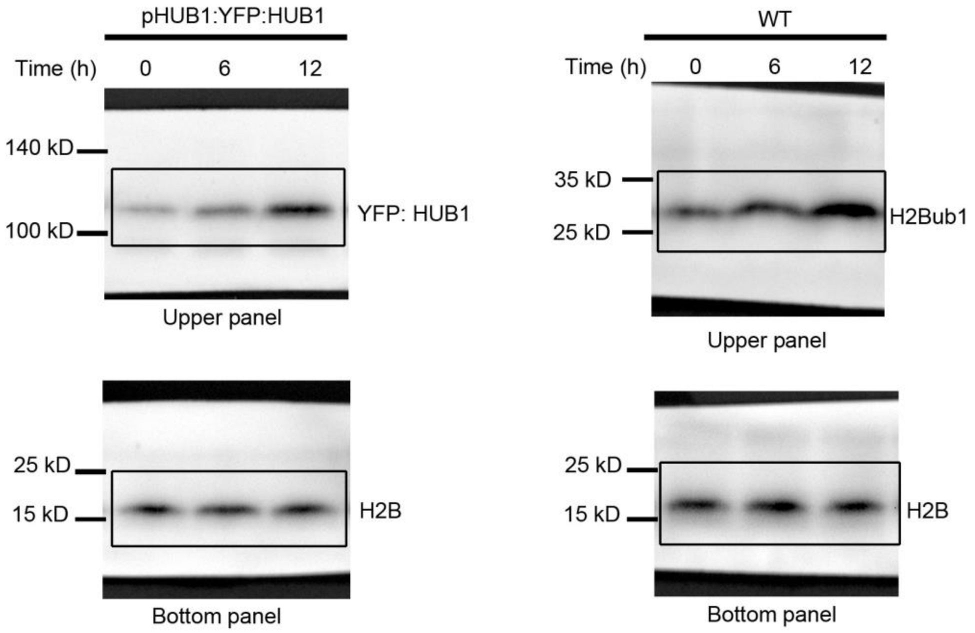

Supplementary Figure 10. Uncropped Western-blots images corresponding to Figure 4b.
