# Supplementary material for: Function of histone H2B monoubiquitination in transcriptional regulation of auxin biosynthesis in Arabidopsis
Source: Commun Biol. 2021 Feb 15;4:206. doi: 10.1038/s42003-021-01733-x (PMC7884795; doi:10.1038/s42003-021-01733-x)
Supplement: Supplementary file 3 — Description of Additional Supplementary Files [file 42003_2021_1733_MOESM3_ESM.pdf]

Description of Additional Supplementary Files

File Name: Supplementary Data 1

Description:
Figure 1 d. Auxin activity in root tips revealed by the expressions of *DII-VENUS*.
Figure 2 c. Similar phenotypes of *ckrw2* and *hub1-5* (b), and molecular complementation.
Figure 3 a. Relative transcription levels of *TSB1*, *WEI7*, *AMI1* and *YUC7* genes in roots.
Figure 3 c. H2Bub1 deposition at specific loci.
Figure 4 a. Effects of 1 μM tZ on CKRW2/HUB1 expression.
Figure 4 d. Comparison of relative expression levels of TSB1 and WEI7 genes between WT and *ckrw2* mutant after tZ treatment.
Supplementary Figure 1. Pleiotropic phenotypes of *ckrw2* mutant, showing significant differences between WT and *ckrw2* in apical hook angle (d), seed size (h), chlorophyll content (i), primary root length (n), length of MZ and MCL (p).
Supplementary Figure 2. Auxin activity in root tips revealed by the expressions of *Dr5: GUS/GFP*, respectively.
Supplementary Figure 3 e. Phenotypic characterization of *ckrw2* and other *hub1* allelic mutants.
Supplementary Figure 6. Expression of related genes in the auxin synthesis pathway.
Supplementary Figure 7. ChIP analysis of H2B monoubiquitin deposition at specific loci.
Supplementary Figure 8. Phenotypic observation and genetic analysis of *ckrw2* and the four of its related mutants (b, c), and the CK-induced HUB1 gene expression (d, e)

File Name: Supplementary Data 2

Description: Primers used in map-based cloning, vector construction, RT-qPCR, and ChIP-qPCR.
